# Ultra high-resolution seawater density sensor based on a refractive index measurement using the spectroscopic interference method

**DOI:** 10.1038/s41598-019-52020-z

**Published:** 2019-10-29

**Authors:** Hiroshi Uchida, Yohei Kayukawa, Yosaku Maeda

**Affiliations:** 1 Research Institute for Global Change, Japan Agency for Marine-Earth Science and Technology, Yokosuka, Japan; 2 National Metrology Institute of Japan, National Institute of Advanced Industrial Science and Technology, Tsukuba, Japan; 3 Institute for Marine-Earth Exploration and Engineering, Japan Agency for Marine-Earth Science and Technology, Yokosuka, Japan

**Keywords:** Marine chemistry, Physical oceanography, Optical sensors

## Abstract

The interference method is one of the most sensitive methods for measuring the refractive index of seawater. We developed a state-of-the-art density sensor for seawater measurements based on measuring the refractive index by the interference method. The resolution of the density sensor is 0.00006 kg/m^3^ for changing temperature at constant salinity and pressure, 0.00012 kg/m^3^ for changing salinity at constant temperature and pressure, and 0.00010 kg/m^3^ for changing pressure at constant temperature and salinity. These resolution values are the best in the history of seawater density measurements. The ultra high-resolution density sensor will contribute notably to climate research at full ocean depth and measurement of seawater sampled from the deep ocean, to research on metrology to establish the traceability of salinity measurements, and to submarine resource exploration to detect spatial changes in the absolute salinity anomaly by combining with conventional conductance-based salinity measurements.

## Introduction

The Earth’s climate system is driven by energy from the sun. Oceanic and atmospheric circulations transfer this energy from the low latitudes of Earth’s surface to higher latitudes. The ocean’s central role in the climate system results from its great capacity to store and transport heat and water and exchange these with the atmosphere. Surface water that cools around Antarctica sinks and forms bottom water, which spreads over the entire global ocean. Recent high-quality hydrographic observations have revealed bottom water warming: 2 mK decade^−1^ in the North Pacific^1^ and around 30 mK decade^−1^ in the Southern Ocean^2^. A supercomputer simulation has shown that surface warming events in the Southern Ocean may cause significant temperature changes in the North Pacific deep water^3^. Ocean salinity is also being recognized as an important indicator of climate change; Antarctic ice-sheet melting is freshening the bottom water around Antarctica^4^. At present, however, even the highest possible quality salinity measurements (uncertainty of about 0.001 g/kg) are not adequate for climate studies in the deep ocean, except for regions close to the sources of deep water^5,6^.

For example, in the western North Pacific along 149°E, the meridional gradients of temperature and salinity in the deep ocean are about 2 mK and 0.0002 g/kg per 100 km, respectively^7^. However, the resolutions of temperature and salinity measurements by the conductivity–temperature–depth (CTD) system (SBE 9*plus*, Sea-Bird Scientific, Bellevue, Washington), which is widely used for high-quality measurements^8^, are 0.2 mK and 0.0004 g/kg, respectively. This means that the spatial resolution of the salinity measurement is 20 times coarser than that of the temperature measurement. In addition, ambiguity of the certified salinity value (±0.001 g/kg) of the International Association of the Physical Sciences of the Ocean (IAPSO) Standard Seawater (SSW) (Ocean Scientific International Ltd., Havant, UK) used to calibrate laboratory salinometers is also a serious problem^9^ that results from the lack of traceability to the International System of Units (SI)^10^. Moreover, absolute salinity, which is needed to calculate the density of seawater from conventional conductance-based salinity measurements with a correction algorithm that uses the global atlas of silicate concentrations in the International Thermodynamic Equation of Seawater 2010 (TEOS-10)^11^, has problems such as the latitude-dependent error induced from the correction algorithm^12^, the effect of high total alkalinity caused by river water in the Arctic Ocean^13^, and the time change of the magnitude of the correction due to the effect of ocean acidification^14^.

Interest in the refractive index-based measurement of density (or absolute salinity) has revived because it could help resolve these problems^15^. In fact, a double-prism V-block refractometer was tested at sea and made commercially available as an absolute salinity sensor^16^, although the pressure resistance (20 MPa) and the resolution of the salinity measurement (0.001 g/kg) are not adequate to detect decadal-time-scale salinity change in the deep ocean. Meanwhile, the interference method is known to be one of the most sensitive ways to measure the index, and it has been reported that the resolution of the salinity measurement (0.0007 g/kg) is better than that of the V-block refractometer^17^, although the sensor has not yet been put to practical use. We therefore developed a practical seawater density (or absolute salinity) sensor based on high-resolution measurement of the refractive index by the spectroscopic interference method that can be used for climate studies in the laboratory and in the deep ocean.

## Results

### State-of-the-art density sensor

**Figure 1:**
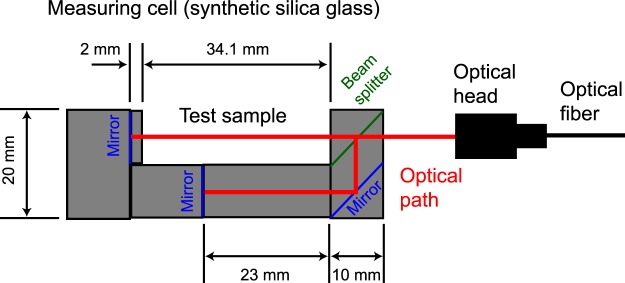
Schematic illustration of the density measuring cell for measurement of the refractive index difference between the test sample and synthetic silica glass. The thickness of the cell is 10 mm. The light beam spot size is about 20 μm. Light from the optical head is split into two paths by a beam splitter. One path goes through the test sample and the other path goes through the glass as a reference optical path. Both light beams reflect at terminal mirrors and return via the original path. The optical head consists only of optical fibers and lenses without electronic parts, which eliminates errors due to heat generation and electromagnetic noise in the measurement.

We developed a seawater density sensor by combining a density measuring cell and a commercially available thickness meter. The density-measuring cell is composed of optical components made from synthetic silicate glass (Fig. 1). The difference between the two optical path lengths (*δX*) can be written as

1 $$\delta X=({X}_{s}{n}_{s}+{X}_{g1}{n}_{g}-{X}_{g2}{n}_{g},)/{n}_{a},$$

where *X*_s_ (34.1 mm) is the length of the test sample, *X*_g1_ (7 mm) is the length of the glass in the optical path for the test sample, *X*_g2_ (38 mm) is the length of the glass in the reference optical path, *n*_s_ is the refractive index of the test sample, *n*_g_ is the refractive index of the synthetic silica glass (about 1.453), and *n*_a_ is the refractive index of air at the calibration of the thickness meter (about 1.00027). We used a spectroscopic interference thickness meter (model SI-F80, Keyence Co., Osaka, Japan) to detect *δX* (sensor output). The measurement principle of the thickness meter is described in Supplementary Information (see Fig. S1). Broad wavelength light (central wavelength of 0.820 μm) is emitted from a superluminescent diode. The interference light of the two reflected light beams from the measuring cell is split into different wavelengths with the spectroscope, and *δX* is determined by waveform analysis (nominal range of *δX* is 50 to 1100 μm with a resolution of 0.001 μm).

### Laboratory test of the density sensor

**Figure 2:**
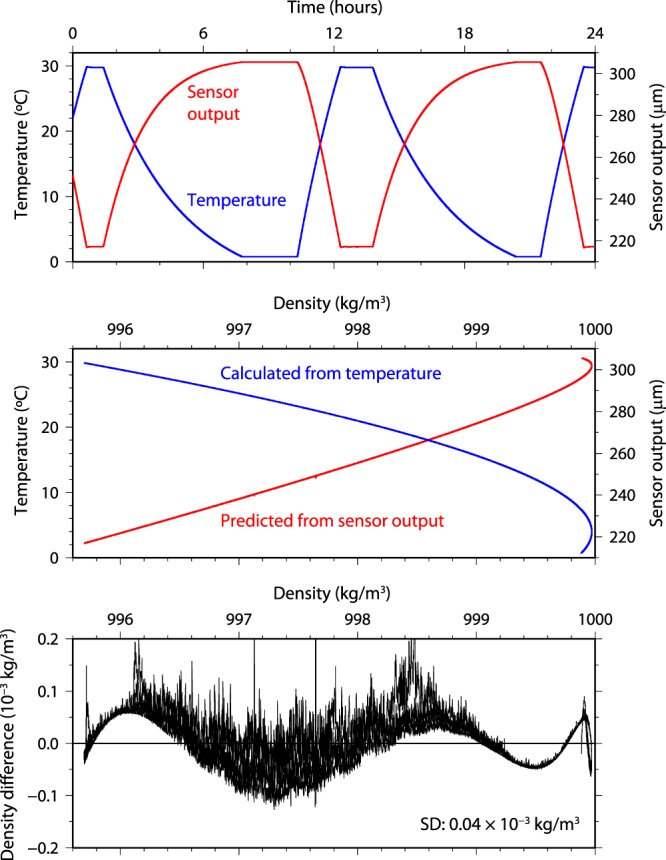
Pure water measurement in the temperature calibration bath. (**a**) Time series of temperature (blue line) of the test sample and sensor output (red line) from the density sensor under atmospheric pressure. The measuring cell (Fig. 1) of the density sensor and the reference thermometer were immersed in pure water in the temperature calibration bath. (**b**) Comparison of densities calculated from the equation of state (TEOS-10) with measured temperature (blue line) and predicted from the sensor output (red line) for all data (86,769 pairs) shown in (**a**). (**c**) Difference between the calculated and predicted densities. The predicted density is estimated from Eq. (2) and the fitting coefficients determined by minimizing the sum of the squares of the differences. The standard deviation of the density difference was 0.04 × 10^−3^ kg/m^3^.

We tested the state-of-the-art density sensor (see Supplementary Information Fig. S2a) in pure water at temperatures from 1 to 30 °C (Fig. 2a). We could easily measure *δX* in the pure water mixed by stirring propellers in the temperature calibration bath. The sensor output changed depending on changes in temperature of the pure water (maximum rate of temperature change was 0.21 °C min^−1^) (the 68% response time was shorter than 1 s; see Supplementary Information Fig. S3). The density of the pure water was predicted from the sensor output and was compared with the density calculated from the equation of state (TEOS-10) to examine the performance of the sensor (precision, short-term stability, repeatability, and temperature hysteresis) for the pure water measurement. To predict the density (*ρ*) from the sensor output, a multivariate (sensor output [*δX*], temperature [*T*], and pressure [*P*]) polynomial curve was fitted to minimize the difference between the calculated density and the predicted density by least squares fitting as follows:

2 $$\begin{array}{rcl}\rho  & = & {C}_{0}+{C}_{1}\,T+{C}_{2}\,{T}^{2}+{C}_{3}\,{T}^{3}+{C}_{4}\,{T}^{4}+{C}_{5}\,{T}^{-1}+{C}_{6}\,\delta X+{C}_{7}\,\delta {X}^{2}+{C}_{8}\,\delta {X}^{3}\\  &  & +{C}_{9}\,\delta X\,T+{C}_{10}\,\delta X\,{T}^{2}+{C}_{11}\,\delta X\,{T}^{3}+{C}_{12}\,\delta X\,{T}^{4}+{C}_{13}\,\delta X\,{T}^{-1}+{C}_{14}\,\delta {X}^{2}\,T\\  &  & +{C}_{15}\,P+{C}_{16}\,{P}^{2}+{C}_{17}\,\delta X\,P+{C}_{18}\,\delta X\,T\,P,\end{array}$$

where *Cx* [*x* = 0–18] are the fitting coefficients, and *P* = 0 here. The predicted density derived from all the sensor outputs followed nearly the same curve (Fig. 2b). The difference between the calculated and predicted densities is shown in Fig. 2c. The precision was estimated to be 0.04 × 10^−3^ kg/m^3^ from the standard deviation of the density difference. The short-term stability was evaluated from the data obtained at constant temperature for 142 min (time from 7.92 to 10.30 hours in Fig. 2a). The means with standard deviations for the temperature and sensor output were 0.7679 ± 0.00048 °C and 305.563 ± 0.0091 μm, respectively. In addition, the means with standard deviation for both densities calculated from the temperature and predicted from the sensor output was 999.88669 ± 0.00003 kg/m^3^. Since the correlation coefficient between the two densities was 0.992, most of the very small variation in the predicted density is explained by the actual density variation due to small temperature variation. Both the repeatability and temperature hysteresis were typically within ± 0.0001 kg/m^3^.

**Figure 3:**
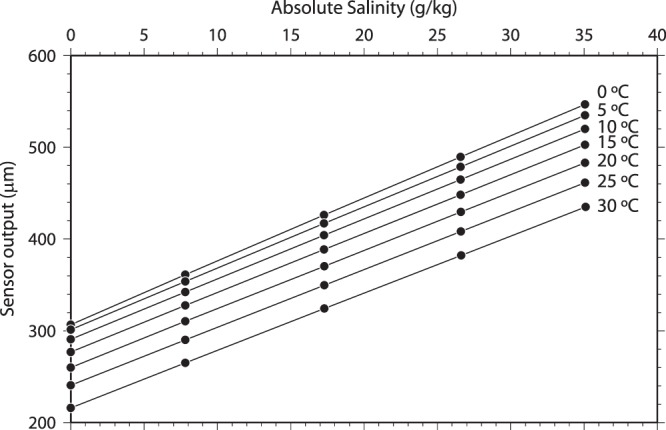
Sensor output plotted against absolute salinity for several temperatures of the test sample. At each salinity and temperature, the temperature and sensor output readings were averaged over 5 min. The SDs of the temperature and sensor output were smaller than 0.3 mK and 0.004 μm, respectively. The absolute salinity was estimated for each test sample at the end of each 5-min measurement by drawing the test sample for salinity analysis.

Similarly, we also tested the density sensor in seawater with absolute salinity (S_A_) values of 7.8, 17.3, 26.6, and 35.1 g/kg. The relationship between S_A_ and the sensor output (*δX*) at temperatures of 0, 5, 10, 15, 20, 25, and 30 °C is shown in Fig. 3. As in previous studies (e.g., ref.^18^), the sensor output showed good linearity with salinity. The sensitivities to the salinity and corresponding density change were evaluated to be 6.79 μm/(g/kg) and 8.48 μm/(kg/m^3^) from the gradients *d*(*δX*)/*d*S_A_ and *d*(*δX*)/*dρ*, respectively, at a temperature of 0 °C. Moreover, the sensitivities to the temperature and corresponding density change were evaluated to be −3.89 μm/°C and 16.70 μm/(kg/m^3^) from the gradients *d*(*δX*)/*dT* and *d*(*δX*)/*dρ*, respectively, at S_A_ of 35 g/kg. Therefore, the resolutions of the salinity and density measurements can be estimated from the sensitivities and the resolution of the sensor output (0.001 μm) and are listed in Table 1.

**Table 1:** Specifications of the state-of-the-art seawater density sensor.

Size	φ 210 mm × 500 mm
Weight	14 kg in air
Depth rating	10,500 m (for pressure-tight housing)
Temperature range	Freezing point −50 °C (must not freeze)
Salinity range	0–120 g/kg @ atmospheric pressure
Pressure range	Atmospheric pressure–120 MPa
Resolution	0.00006 kg/m^3^ @ 35 g/kg (for changing temperature) 0.00012 kg/m^3^ (0.00015 g/kg) @ 1 °C (for changing salinity) 0.00010 kg/m^3^ @ 35 g/kg (for changing pressure)
Sampling frequency	5000 Hz (for practical measurement resolution of 0.01 kg/m^3^) 1.2 Hz (for practical measurement resolution of 0.0001 kg/m^3^)

**Figure 4:**
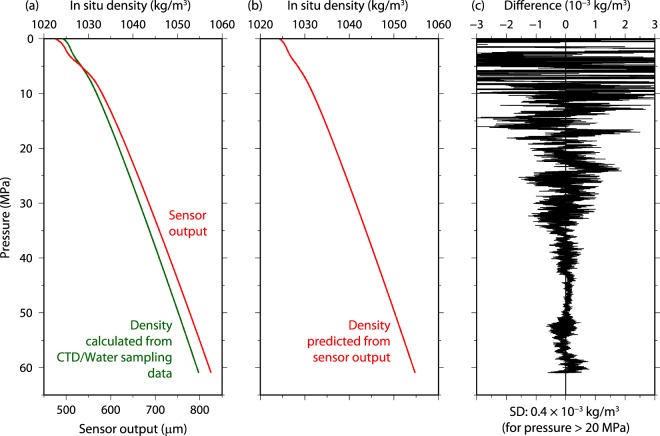
Seawater measurement in the deep ocean of the North Pacific (32.2°N, 144.3°E). (**a**) Vertical profiles of *in situ* density (green line) calculated from CTD/water sampling data and output (red line) from the density sensor. (**b**) Vertical profile of *in situ* density predicted from the density sensor output. (**c**) Difference between the calculated and predicted densities. The predicted density is estimated from Eq. (2), and the fitting coefficients determined by minimizing the sum of the squares of the differences. The standard deviation of the density difference was 0.4 × 10^−3^ kg/m^3^ for pressure greater than 20 MPa.

Size and weight are approximate. Temperature range was limited by the manufacturer’s specifications for the spectroscopic interference thickness meter. Salinity range was estimated from the limited laboratory experiment at atmospheric pressure (Fig. 3) by extrapolating to both ends of the measurement range (50–1100 μm) of the thickness meter. Pressure range was estimated from the limited field experiment (Fig. 4) by extrapolating to both ends of the measurement range of the thickness meter. The resolution was estimated from the sensitivity to salinity change (6.79 μm/[g/kg]) and corresponding density change (8.48 μm/[kg/m^3^]) at constant temperature and pressure, and changing temperature (−3.89 μm/°C) at constant salinity and pressure estimated from the laboratory experiment and the resolution of the sensor output (0.001 μm). The resolution for changing pressure was also estimated from the sensitivity to pressure change (4.4 μm/MPa) at nearly constant temperature and salinity (deep ocean). To achieve finest measurement resolution of 0.001 μm, the sensor output will have to be averaged over 0.82 s (4096 data points).

### Field test of the density sensor

We tested the density sensor in the North Pacific (up to 60 MPa) with the CTD/water sampling system of the RV *Mirai* in May 2019 (see Supplementary Information Fig. S2b). The sensor output changed depending on changes in pressure similarly to the *in situ* density calculated from the equation of state (TEOS-10) (Fig. 4a). To predict the *in situ* density from the sensor output, a multivariate polynomial curve (Eq. 2) was used to minimize the difference between the calculated density and the predicted density by least squares fitting (Fig. 4b). The difference between the calculated and predicted densities is shown in Fig. 4c. The standard deviations of the density differences were determined to be 0.0004 and 0.0038 kg/m^3^ for pressures greater than and smaller than 20 MPa, respectively. To the best of the authors’ knowledge, this was the first refractive index-based measurement of *in situ* seawater density in the deep ocean (pressure greater than 20 MPa).

## Discussion

### Determination of the density from the density sensor

In principle, the refractive index of the test sample can be determined from Eq. (1). In practice, however, it is not easy to precisely quantify the refractive index of the glass and the lengths of the glass and test sample. Therefore, it is practical to directly estimate the density of the test sample from the sensor output. The empirical equation to predict the density should be a function of the sensor output, temperature, and pressure.

Next, we tried to estimate the density from the sensor output by using the results of the laboratory test of pure water and seawater measurements (Fig. 3). The multivariate (sensor output, temperature, and pressure) polynomial curve (Eq. 2) was fitted to the sensor output by the least squares method. The standard deviation of the density difference between the calculated and predicted densities was larger (0.0011 kg/m^3^) than the result for only pure water (0.00004 kg/m^3^, Fig. 2c). Although the residual may be smaller than the value in a previous study (0.0035 kg/m^3^)^19^, it still must be reduced. Possible sources of this large residual are (1) insufficient empirical function used to predict the density, (2) error caused by nonlinearity of the spectroscopic interference thickness meter, and (3) error in the equation of state of seawater (TEOS-10). The second possible source seems likely because similar undulations of the density difference were seen in the laboratory and in the deep ocean (Figs 2c and 4c), although such undulations were not present in one portion of Fig. 4c (e.g., pressure from 42.5 to 50 MPa or sensor output from 744 to 777 μm). These undulations might be caused by a nonlinearity of the thickness meter. The third possible source would be a serious problem and is discussed below.

When using the density sensor in the laboratory at constant temperature and pressure (atmospheric pressure), the calibration equation can be expressed as a function of only the sensor output. Because the sensor output shows good linearity with salinity (Fig. 3), the density sensor could be more precisely calibrated than the *in situ* sensor. In other words, the salinity of seawater sampled from the deep ocean could be measured more precisely in the laboratory than in the deep ocean for climate studies free from the effects of temperature and pressure changes.

### Density error of TEOS-10

Uncertainty in the density dataset for standard seawater used in TEOS-10 is estimated to be 0.004 kg/m^3^ at atmospheric pressure for salinities up to 42 g/kg and for temperatures up to 40 °C^20^. In addition, a recent density measurement of standard seawater by means of a vibrating-tube density meter suggests that the density calculated from TEOS-10 increasingly deviates with salinity, with the increase approximately linear and about 0.015 kg/m^3^ at salinity of 35 g/kg^21^. This result is supported by their density measurement by means of hydrostatic weighing^22^. However, another study reported a smaller density deviation (0.0064 kg/m^3^) based on a vibrating-tube density meter^12^, and our recent results of density measurements by means of hydrostatic weighing showed almost no deviation. A way to resolve this discrepancy would be to evaluate the density of standard seawater as accurately as possible by means of hydrostatic weighing as the primary standard method, preferably by multiple institutions.

### *In situ* density sensor

Typically, turbidity and sunlight can affect optical measurements of the density sensor in the surface ocean (~100 m), although optical measurements in the deep ocean are free from these effects. Density non-uniformity in a test sample along the optical path (34.1 mm) of the measuring cell may occur due to oceanic temperature and salinity microstructures. However, the density sensor measures the average of the refractive index of the test sample along the optical path. In addition, the effect of temperature and salinity microstructures might be removed by averaging the obtained data in time and space, similar to a standard CTD observation (e.g., temperature and salinity vertical profiles are usually averaged at an interval of 1 or 2 dbar in climate studies).

The sensitivity to pressure change (4.4 μm/MPa) in the deep ocean (Fig. 4) is mainly due to the *in situ* density change and therefore change of *n*_s_ in Eq. (1), but it may also be due to pressure dependency of the measurement cell (a change of geometry [*X*_s_, *X*_g1_ and *X*_g2_ in Eq. 1] and a change of refractive index of the synthetic silica glass [*n*_g_ in Eq. 1]). It is difficult to separate these effects from the sensor output. Therefore, we empirically predicted the density from the sensor output by the polynomial curve (Eq. 2).

We will also test the density sensor in the Izu-Ogasawara Trench (~9300 m) with the CTD/water sampling system of the RV *Kaimei*^23^. The hadal zone (depths greater than around 6000 m) in ocean trenches is one of the last frontiers in the global ocean^24^. Density measurements *in situ* and for water samples in the trench should be suitable for more precise evaluation of the pressure dependency of the density sensor because potential density profiles obtained in the trench are reasonably homogeneous^23^.

### Comparison with the de facto standards

We compared the density measurement resolution of the state-of-the-art density sensor with those of the de facto standard salinometer, densimeter, and refractometer. The resolution of the laboratory conductivity salinometer (Autosal 8400B, Guildline Instruments Ltd., Ontario, Canada) is 0.00015 kg/m^3^ (0.0002 g/kg in salinity in the range of 0.005–42 g/kg). The resolution of the *in situ* conductivity sensor (SBE 4, Sea-Bird Scientific) used in the SBE 9*plus* CTD system is 0.00030 kg/m^3^ (0.0004 g/kg in salinity). The resolution of the laboratory densimeter (DMA 5000 M, Anton-Paar GmbH, Graz, Austria) is 0.001 kg/m^3^. The resolution of the brand-new and commercially available refractometer (NOSS sensor, nke Instrumentation, Hennebont, France) is 0.0008 kg/m^3^ (0.001 g/kg in salinity in the range of 15–42 g/kg)^25^. To our knowledge, the resolution of the density sensor developed in this study (0.00012 kg/m^3^, Table 1) is the best in the history of seawater density measurements.

### Future view

The state-of-the-art density sensor can measure seawater density with better resolution than the de facto standard *in situ* and laboratory conductivity salinometers and one order of magnitude finer than the de facto standard laboratory densimeter and *in situ* refractometer over a wider range of salinity (0–120 g/kg) (see Supplementary Information Fig. S5). It is an important milestone to substitute the method of salinity measurement in the ocean from one that is conductance-based and has been used for more than 40 years to the index-based method, which can directly estimate seawater density.

We will be able to use the ultra high-resolution density sensor in research on, for example, global climate change to detect deep ocean freshening associated with global warming and Antarctic ice-sheet melting, metrology to establish the traceability of the salinity of IAPSO SSW to SI units with small uncertainty, and submarine resource exploration to detect spatial changes in the absolute salinity anomaly associated with submarine springs in real time by combining the density sensor with a conventional conductivity sensor.

## Methods

### Laboratory test

We measured test samples (pure water and seawater) by using the state-of-the-art density sensor under atmospheric pressure. The density-measuring cell and a reference thermometer (model SBE 35, Sea-Bird Scientific) were immersed in a refrigerated temperature calibration bath (model 7011, Fluke Co., Everett, Washington). The temperature of the test sample (about 27 L) near the measuring cell was measured by the reference thermometer at a sampling interval of about 3.6 s and interpolated at an interval of 1 s. The expanded uncertainty of the temperature measurement can be estimated to be 0.4 mK^26^. The difference between the two optical path lengths (*δX*) was measured at a sampling interval of 0.0002 s and was low-pass-filtered with a half power gain at 10 s, and the sensor output was stored at an interval of 1 s and merged with the temperature data. The spectroscopic unit of the thickness meter was kept at room temperature (around 23 °C) or put into an incubator to maintain the temperature of the unit at about 24 °C. The room temperature and temperature in the incubator were measured by a laboratory thermometer (model 1502 A, Fluke Co.) at an interval of 1 s to correct for temperature dependency of the spectroscopic unit. The density error corresponding to the temperature dependency was estimated to be 0.0015 [kg/m^3^]/°C (see Supplementary Information Fig. S4).

We used ultra-pure water prepared from Yokosuka, Japan, tap water by using an ultra purification water system (model UL-Pure KE0196, Komatsu Electronics Co., Ltd., Komatsu, Ishikawa, Japan), and pure water (Pure Water [water hardness 0], Ako Kasei Co., Ltd., Ako, Hyogo, Japan) made from seawater collected from a depth of 344 m off Muroto, Kochi, Japan, by filtering twice with a reverse osmosis membrane. In addition, Pure Water was deionized using ion exchange resin (Pure Maker, Sanei Corp., Arao, Kumamoto, Japan). We also used Atlantic Seawater (Ocean Scientific International Ltd.) and Atlantic Seawater diluted with Pure Water. The Atlantic Seawater is the source seawater of IAPSO SSW. These test samples were installed in the temperature calibration bath and measured with the density sensor.

We calculated the densities of the test samples by using TEOS-10. For ultra-pure water, the effect of the isotopic composition^27^ for hydrogen (*δD*) and oxygen (*δ*^18^*O*) relative to Vienna Standard Mean Ocean Water (VSMOW) was corrected by using typical values for Yokosuka tap water (*δD* of −59‰ and *δ*^18^*O* of −9‰, e.g., ref.^12^) (Fig. 2). For the Pure Water, the effect of the isotopic composition was corrected by using measured values (*δD* of −3.4‰ and *δ*^18^*O* of −1.3‰) (Fig. 3). The practical salinity measured for the ultra-pure water and Pure Water was 0.0000 and 0.0003, respectively, although these values were outside the warranty range (0.005–42 in practical salinity) of the salinometer (Autosal 8400B). The absolute salinity was estimated for each seawater sample and temperature by drawing the seawater sample into a 100 mL bottle for practical salinity analysis. The practical salinity was measured with a salinometer (Autosal 8400B), which was standardized with IAPSO SSW batch P160. The absolute salinity anomaly was assumed to be zero for the seawater sample.

### Field test

To be able to use the density sensor at full ocean depth, the spectroscopic and control units (about 130 mm high, 130 mm wide, and 130 mm long) are put inside a cylindrical pressure-tight housing using a ceramic cylinder (A479, Kyocera Co., Fushimi, Kyoto, Japan) and aluminum alloy (A7075-T6) hemispherical caps^28^ to reduce weight and cost compared to a titanium alloy housing (see Supplementary Information Fig. S2b). The specifications of the density sensor as designed and as evaluated from the laboratory and field tests are summarized in Table 1. The temperature in the pressure-tight housing was measured by a temperature logger (model Duet T.D. deep, RBR Ltd., Ottawa, Canada) at an interval of 1 s to correct for the temperature dependency of the spectroscopic unit. The density sensor output was low-pass-filtered with a half power gain at 1 s, and was stored at an interval of 0.0217 s through the Serial Data Uplink of the CTD/water sampling system (SBE 9*plus*, Sea-Bird Scientific).

The density sensor was used with the CTD/water sampling system. The CTD data obtained at an interval of 0.0417 s were averaged at an interval of 1 s. The CTD thermometer was calibrated with the reference thermometer (SBE 35)^26^, and the CTD salinity data were corrected by using salinity data measured for the *in situ* water sample data. Salinity measurements for the water samples were conducted with a salinometer (Autosal 8400B). The absolute salinity anomaly profile was estimated from the discrete water sample data (silicate, nitrate, total alkalinity, and dissolved inorganic carbon)^29^ by using an Akima spline interpolation. We calculated the reference densities from the CTD and absolute salinity anomaly data by using TEOS-10. The reference density data were merged with the density sensor output and averaged at an interval of 1 s. Down-cast profiles were used for the analysis.

## Supplementary information

Ultra high resolution seawater density sensor based on a refractive index measurement using the spectroscopic interference method
