# Supplementary material for: Ultra high-resolution seawater density sensor based on a refractive index measurement using the spectroscopic interference method
Source: Sci Rep. 2019 Oct 29;9:15482. doi: 10.1038/s41598-019-52020-z (PMC6820790; doi:10.1038/s41598-019-52020-z)
Supplement: Supplementary file 1 — Ultra high resolution seawater density sensor based on a refractive index measurement using the spectroscopic interference method [file 41598_2019_52020_MOESM1_ESM.pdf]

Supplementary Information

Ultra high-resolution seawater density sensor based on a refractive index measurement using the spectroscopic interference method

Hiroshi Uchida[1], Yohei Kayukawa[2], and Yosaku Maeda[3]

[1]Research and Development Center for Global Change, Japan Agency for Marine-Earth Science and Technology

[2]National Metrology Institute of Japan, National Institute of Advanced Industrial Science and Technology

[3]Marine Technology and Engineering Center, Japan Agency for Marine-Earth Science and Technology

Measurement principle of the spectroscopic interference thickness meter

A schematic of the measurement principle of the spectroscopic interference thickness meter (model SI-F80, Keyence Co., Osaka, Japan) is shown in Fig. S1 based on the manufacturer's brochure. Broad wavelength light (0.820-μm central wavelength) emitted from a superluminescent diode (SLD) travels in a polarization-maintaining optical fiber and emerges into air from the optical head. Part of the emitted light is reflected by the top surface of the target object (e.g., glass), while the part that passes the top surface is reflected by the bottom surface of the target object. The two reflected light beams interfere with each other and return into the optical head. The interference light is split into different wavelengths by a diffraction grating spectroscope. The optical intensity of the interference light has periodicity $cos(2\pi \times 2\delta X/\lambda)$, where δX is the difference between the two optical path lengths and λ is the wavelength of the light. The δX is obtained by a fast Fourier transform (FFT) waveform analysis of the optical intensity distribution by λ taken by the charged-coupled devices (CCD) as the thickness of the target object.

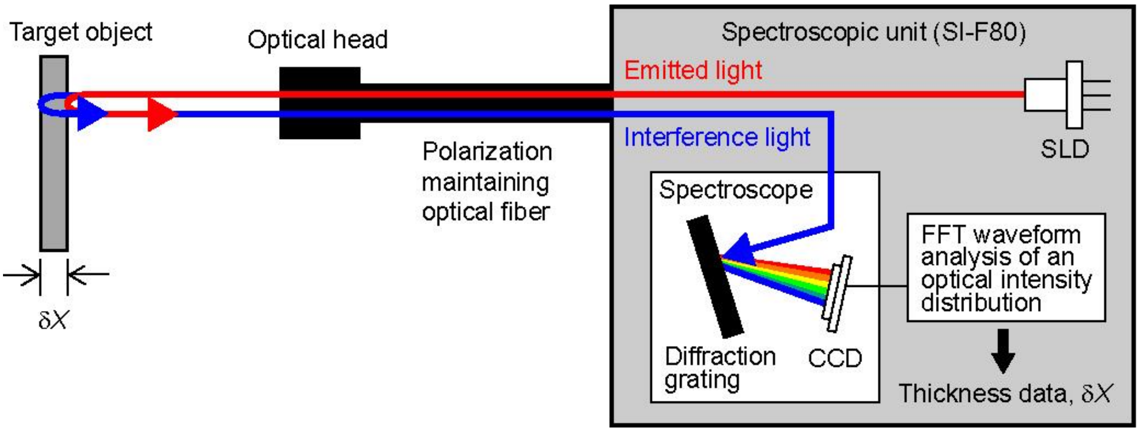

Figure S1. Schematic illustration of measurement principle of the spectroscopic interference thickness meter.

Photos of the density sensors

In the laboratory test, the measuring cell with a reference thermometer (SBE 35, Sea-Bird Scientific, Bellevue, Washington) was immersed in a temperature calibration bath (model 7011, Fluke Co., Everett, Washington) (Fig. S2a). The spectroscopic unit of the interference thickness meter was put into an incubator to maintain the temperature of the unit at about 24 °C. The controller of the spectroscopic unit (model SI-F1000, Keyence Co.) was kept at room temperature of around 23 °C. In the field test, the

measuring cell was attached to the synthetic silicate glass window of the pressure-tight housing (Fig. S2b). The spectroscopic unit and controller were put into the pressure-tight housing. The temperature and pressure in the pressure-tight housing were measured by a temperature and pressure logger (model Duet T.D. deep, RBR Ltd., Ottawa, Canada) to correct for temperature dependency of the spectroscopic unit. The pressure in the pressure-tight housing was set to around 700 hPa by depressurization to secure the cylindrical pressure-tight housing and hemispherical caps. The density sensor was used with a conductivity-temperature-depth (CTD) system (SBE 9*plus*, Sea-Bird Scientific, Bellevue, Washington).

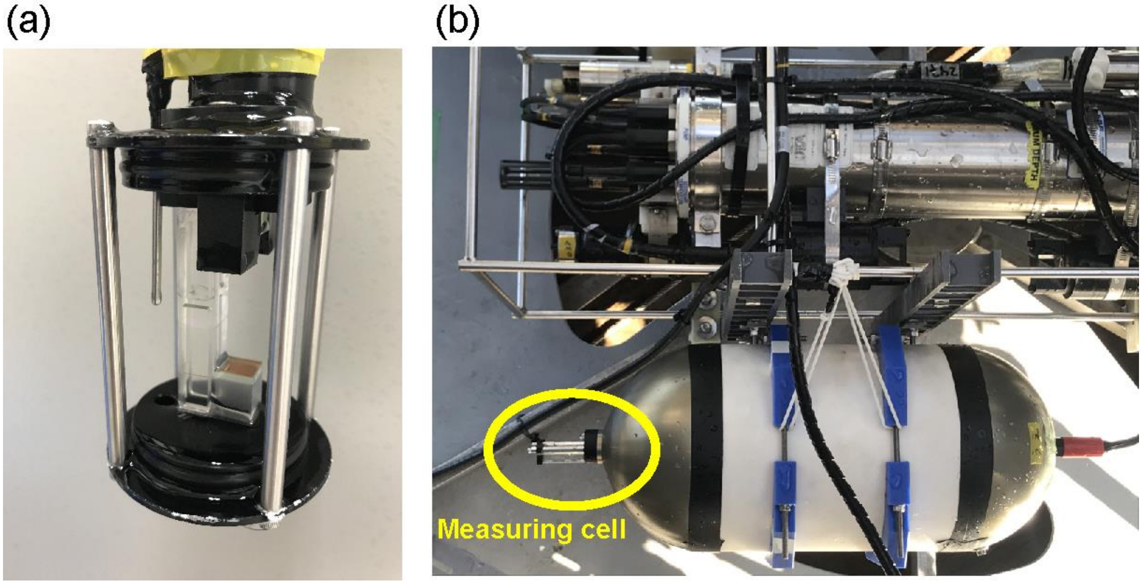

Figure S2. Photos of the density sensors (a) in the laboratory and (b) in the field with a CTD system (top view).

Response time

The response time of the density sensor against temperature change of a test sample was examined in the laboratory. Two temperature calibration baths were filled with pure water. The baths were set to temperatures of 1 °C and 30 °C, respectively. The measuring cell was immersed in one bath until the sensor output stabilized, and then the measuring cell was quickly moved and immersed in the other bath (Fig. S3). The response times of the sensor were nearly the same for the temperature increase and decrease cases, it was almost shorter than 1 s to reach 68% and about 12 s to reach 95% of the expected change in the sensor output for the test sample.

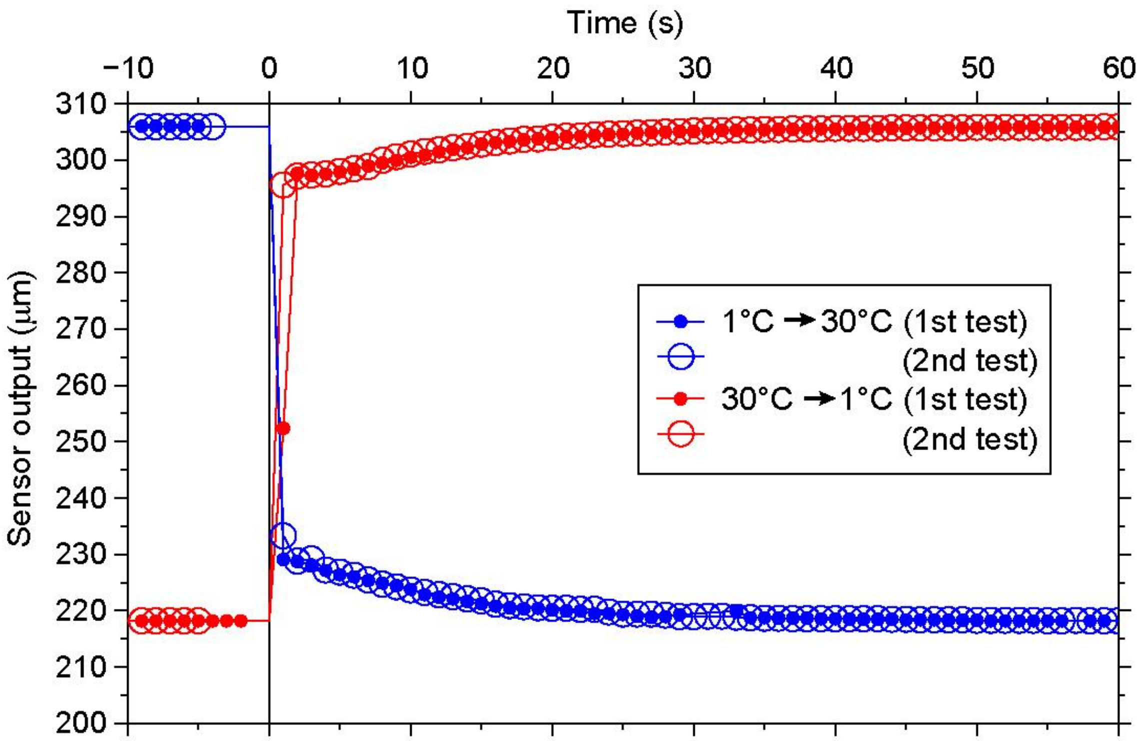

Figure S3. Time series of the sensor output against quick changes in temperature of a test sample in pure water.

Temperature dependency of the spectroscopic unit

The sensor output of the thickness meter changes in response to temperature changes of the spectroscopic unit. The temperature dependency of the spectroscopic unit was examined by a laboratory test. The spectroscopic unit was put into an incubator inside of which the temperature was measured by a laboratory thermometer (model 1502A, Fluke Co.). The measuring cell of the density sensor and the reference thermometer were immersed in pure water in the temperature calibration bath. The sensor output was obtained at test sample (pure water) temperatures of 2.93, 10.13, 19.96, and 29.77 °C by changing the temperature of the spectroscopic unit to 5, 11, 21, and 31 °C, respectively. For each temperature of the test sample, the sensor output at the spectroscopic unit temperature of 24 °C was estimated by second-order polynomial curve fitting; the change in the sensor output from the value at 24 °C is shown in Fig. S4. The change in the sensor output ($\Delta\delta X$) is slightly dependent on the temperature of the test sample (T), hence $\Delta\delta X$ is modeled as

$$\Delta\delta X = (c_0 + c_1 T)\,\Delta t + (c_2 + c_3 T)\,(\Delta t)^2, \quad \text{(S1)}$$

where Δt is the temperature anomaly of the spectroscopic unit from 24 °C and cx [x = 0−3] are fitting coefficients. The fitting coefficients were determined by the least squares method. The sensor output corrected for the temperature dependency of the spectroscopic unit ($\delta Xcor$) is written as

$$\delta Xcor = \delta X - \Delta\delta X. \quad \text{(S2)}$$

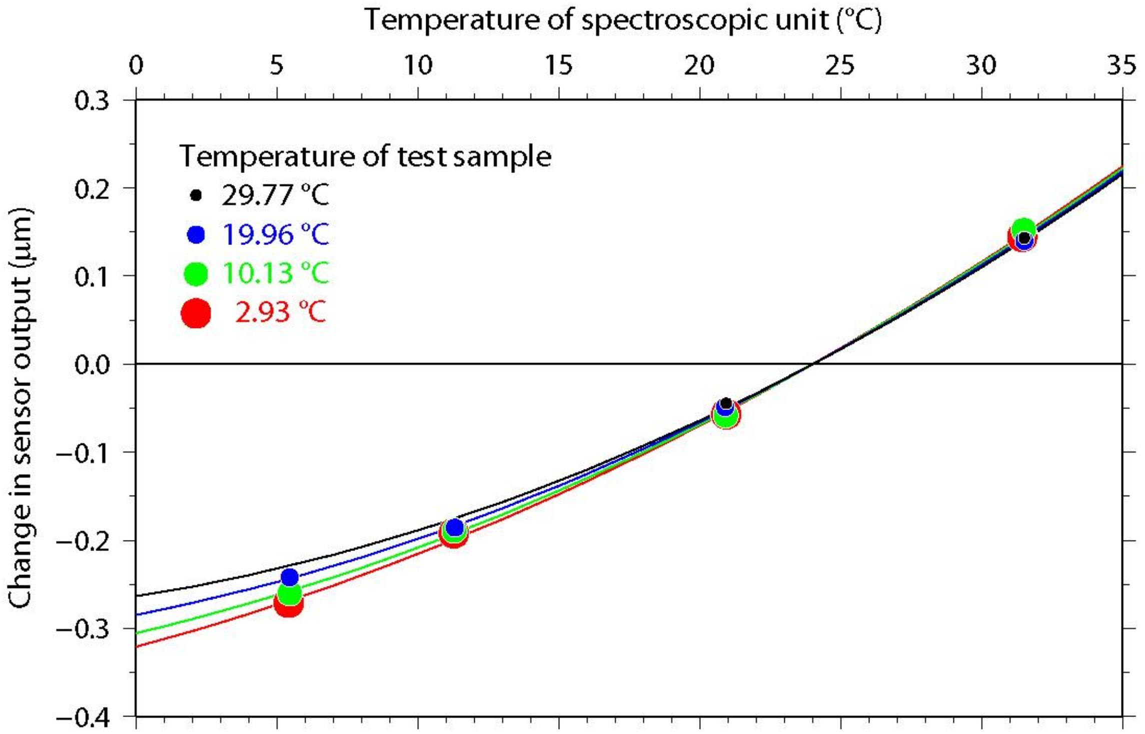

Figure S4. Temperature dependency of the spectroscopic unit. The curves are the predictions from Eq (S1) at each temperature of the test sample.

Measurement of high salinity sample

To confirm the estimated measurement range of salinity (0–120 g/kg @ atmospheric pressure), a high-salinity sample was measured in the laboratory. The high-salinity sample was prepared from International Association of the Physical Sciences of the Ocean (IAPSO) Standard Seawater (SSW) (Ocean Scientific International Ltd., Havant, UK) by increasing the concentration of salinity to about 118 g/kg by water evaporation in an automatic oven. The measuring cell and the reference thermometer were put in a plastic holder filled with the high-salinity sample, and the plastic holder was immersed in a temperature calibration bath (Fig. S5). Absolute salinity (118.032 g/kg) of the

high-salinity sample was back calculated from the density measured at 20 °C by using a vibrating tube density meter (DMA 5000M, Anton-Paar GmbH, Graz, Austria) with the International Thermodynamic Equation of Seawater 2010 (TEOS-10). We can confirm that the density sensor can measure salinity of 120 g/kg for temperatures higher than about 5 °C from the results shown in Fig. S5, the sensitivity of the density sensor to salinity change (6.79 µm/[g/kg]), and the measurement range (50–1100 µm) of the thickness meter.

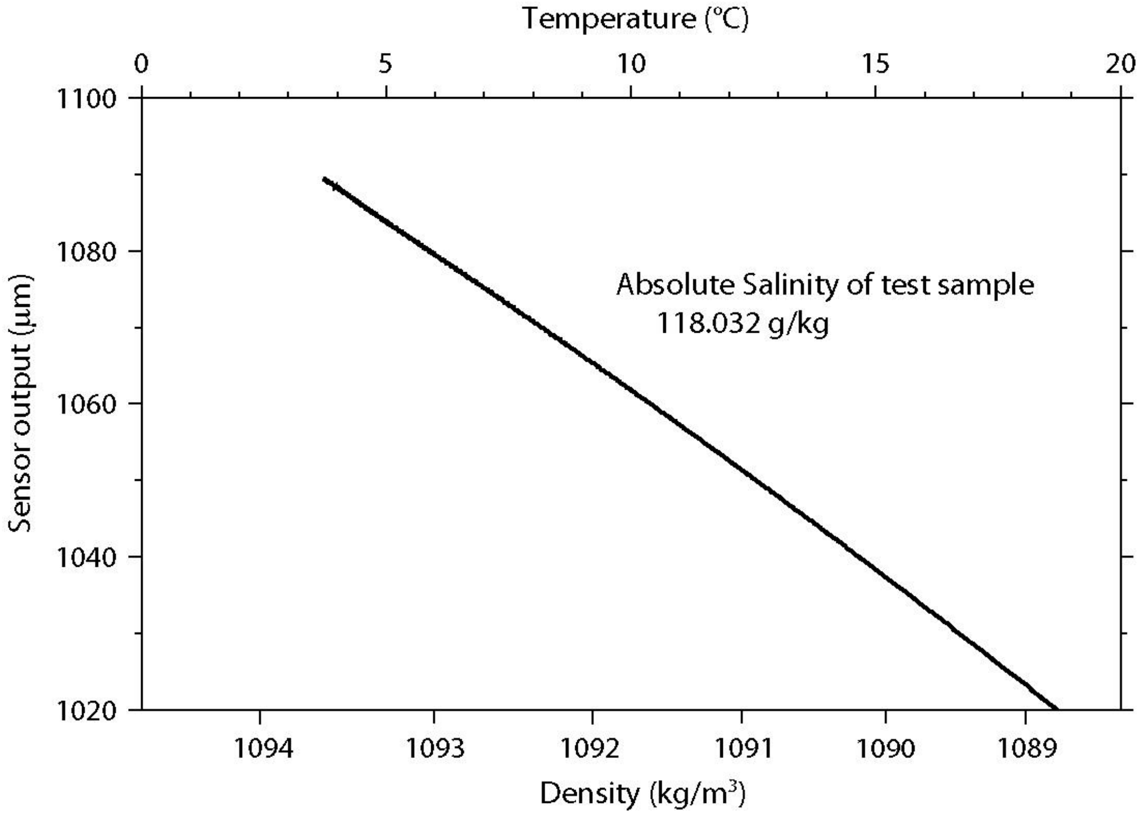

Figure S5. Comparison of sensor output and temperature for the high-salinity sample. The density calculated from the equation of state (TEOS-10) with the measured temperature and the absolute salinity is also indicated.
